# A cuproptosis and copper metabolism–related gene prognostic index for head and neck squamous cell carcinoma

**DOI:** 10.3389/fonc.2022.955336

**Published:** 2022-08-22

**Authors:** Shuaiyuan Zhang, Lujin Zhang, Huanzi Lu, Yihuan Yao, Xiaoyong Liu, Jingsong Hou

**Affiliations:** ^1^ Department of Oral and Maxillofacial Surgery, Guanghua School of Stomatology, Hospital of Stomatology, Sun Yat-sen University, Guangzhou, China; ^2^ Guangdong Provincial Key Laboratory of Stomatology, Sun Yat-sen University, Guangzhou, China

**Keywords:** cuproptosis, copper metabolism-related genes, gene signature, nomogram, prognosis, head and neck squamous cell carcinoma

## Abstract

**Background:**

The purpose of this study was to identify the prognostic value of cuproptosis and copper metabolism–related genes, to clarify their molecular and immunological characteristics, and to elucidate their benefits in head and neck squamous cell carcinoma (HNSCC).

**Methods:**

The details of human cuproptosis and copper metabolism–related genes were searched and filtered from the msigdb database and the latest literature. To identify prognostic genes associated with cuproptosis and copper metabolism, we used least absolute shrinkage and selection operator regression, and this coefficient was used to set up a prognostic risk score model. HNSCC samples were divided into two groups according to the median risk. Afterwards, the function and immune characteristics of these genes in HNSCC were analyzed.

**Results:**

The 14-gene signature was constructed to classify HNSCC patients into low-risk and high-risk groups according to the risk level. In the The Cancer Genome Atlas (TCGA) cohort, the overall survival (OS) rate of the high-risk group was lower than that of the low-risk group (P < 0.0001). The area under the curve of the time-dependent Receiver Operator Characteristic (ROC) curve assessed the good performance of the genetic signature in predicting OS and showed similar performance in the external validation cohort. Gene Ontology and Kyoto Encyclopedia of Genes and Genomes enrichment assays and Protein-Protein Interaction (PPI) protein networks have been used to explore signaling pathways and potential mechanisms that were markedly active in patients with HNSCC. Furthermore, the 14 cuproptosis and copper metabolism-related genes were significantly correlated with the immune microenvironment, suggesting that these genes may be linked with the immune regulation and development of HNSCC.

**Conclusions:**

Our results emphasize the significance of cuproptosis and copper metabolism as a predictive biomarker for HNSCC, and its expression levels seem to be correlated with immune- related features; thus, they may be a possible biomarker for HNSCC prognosis.

## Introduction

Head and neck squamous cell carcinoma (HNSCC) originates from the epithelial cells of the mouth, pharynx, and larynx, which is a kind of malignant tumor with high incidence (1, 2). Smoking, alcohol consumption, viral infection, environmental pollutants, and genetics are all risk factors for HNSCC (1). Since the beginning of this century, great progress has been made in the research of immunotherapy and other targeted drugs related to HNSCC, which have been applied in clinic. However, the five-year survival rate of HNSCC is only 50% (3, 4). In addition, due to the asymptomatic character of HNSCC and the lack of early detection, the risk of recurrence and metastasis of cancer patients is still high (5). Therefore, it is particularly critical to uncover new biomarkers for HNSCC patients and to better customize the prevention, screening, and treatment of HNSCC.

Copper is a trace element that has a significant impact on human health. It exists in organisms in the form of Cu (I) and Cu (II). Existing experimental results show that copper plays an important role in being an intracellular antioxidant, cellular respiration, nerve signal transduction, and the development of extracellular matrix (6). However, at high concentrations, copper can also cause the destruction of intracellular lipids, proteins, and nucleic acids (7). Unlike the known mechanisms of cell death, copper-dependent cell death depends on mitochondrial respiration, which occurs through the direct combination of copper with the fatty acylation component of the tricarboxylic acid (TCA) cycle (8). At present, some antitumor drugs can specifically induce cancer cell death, but how to overcome drug resistance is still an important problem to be solved. Platinum-based antitumor drugs (cisplatin and carboplatin) are commonly used to treat multiple types of solid tumors, including HNSCC. However, there is sufficient evidence to suggest that the long-term use of platinum-based antitumor agents in HNSCC patients can develop drug resistance (9–11). Cuproptosis and copper metabolism may be beneficial to the treatment of drug-resistant HNSCC. Copper homeostasis imbalance can be observed in the progression of HNSCC, and copper metabolism–related genes are related to the sensitivity of HNSCC to platinum compounds (9, 12). Copper can enhance the sensitivity of HNSCC patients to cisplatin by combining with copper ionophore disulfide (13). Therefore, it is very important to explore new forms of regulatory cell death, such as cuproptosis, and to find practical and effective prognostic markers for HNSCC. Thus, Tsvetkov et al. suggest that there may be a window that allows an increased concentration of copper in cells to be used to selectively kill cancer cells (14). The activation of the copper-induced cell death pathway may surpass the current drug resistance of chemotherapeutic drugs and open a new research field for new tumor therapies. At present, researchers have great interest in the relationship between cuproptosis and tumor, and its function in HNSCC needs to be further clarified (15–18). Therefore, the role of the entire cuproptosis and copper metabolism–related gene repertoire, in the prognosis of HNSCC, needs to be investigated.

In this study, HNSCC transcripts from several patient cohorts were downloaded from the public database, and 14 genetic signatures of cuproptosis and copper metabolism were identified by bioinformatics analysis. These markers related to cuproptosis and copper metabolism may become an effective index for predicting the prognosis of HNSCC patients

## Method

### Data collection

Through a comprehensive analysis of the source literature, we screened 139 genes related to cuproptosis and copper metabolism from the msigdb database and added recently reported genes to integrate them for the next step. The TCGA database was used to obtain patient clinicopathological data (https://portal.gdc.cancer.gov/). The data of 546 HNSCC samples were downloaded, including 502 cancer samples and 44 precancerous samples. All 44 adjacent normal tissue specimens belong to the normal group. The phenotype information, survival data (version: 12-06-2021), and RNA sequence data (version: 12-06-2021) of HNSCC patients, cervical cancer Cervical squamous cell carcinoma andendocervical adenocarcinoma (CESC) patients and esophageal cancer (ESCA) patients were acquired from the public database UCSC Xena (https://xenabrowser.net/datapages/). Those without RNA-seq data were excluded.

### Construction and validation of the cuproptosis and copper metabolism–related gene prognostic index

Univariate Cox regression analysis (P<0.05) was applied to sift prognostic-related genes; 35 genes were identified, and 14 differentially related genes were identified by least absolute shrinkage and selection operator (LASSO) regression. Complete the above steps through the glmnet package. The coefficient (β) of LASSO regression corresponding to each gene was performed to construct the risk score for predicting the prognosis. Risk score = (coefficient Gene1 × expression of Gene1) + (coefficient Gene2 × expression of Gene2) +……+ (coefficient Gene n × expression Gene n), and the intermediate value of risk score is used to distinguish high-risk groups from low-risk groups. The prognostic power of the index was assessed by Kaplan-Meier (K-M) survival curves with log-rank tests. In order to test the independent prognostic accuracy of the index, we established 1-year, 3-year and 5-year time-dependent ROC charts, analyzed them with survival ROC software, and calculated the area under the curve (AUC).

### Differentially expressed gene identification and bioinformatics analysis

The Wilcoxon function in R is used to calculate differentially expressed genes (DEGs) between clustering subgroups False discovery rate (FDR<0.05 and |log2FC|>1). According to all the data of DEGs, Gene Ontology (GO) and Kyoto Encyclopedia of Genes and Genomes (KEGG) analyses were studied. Then, the function enrichment analysis of DEGs protein–protein interaction network was fulfilled through the Search tool for the retrieval of interacting genes/proteins (STRING) database (https://string-db.org/), and the online calculation and prediction were carried out. On this basis, the visibility of PPI is analyzed by using Cytoscape. Principal component analysis (PCA) was used for realizing the dimensionality reduction and visualization of signature genes. In addition, the potential biological functions were studied by gene set enrichment analysis (GSEA 4.1.0), and P < 0.05 and FDR < 0.05 were considered statistically significant.

### Tumor immune microenvironment analysis

In order to clarify the prognostic characteristics of the tumor immune microenvironment, the high-risk group and low-risk group were compared. We applied the CIBERSORTx website (https://cibersortx.stanford.edu/) to compare and reckon the abundance of 22 immune cells in the two populations. Platform xCell (xCell signature) (19) was used to calculate the proportion of immune cell infiltration in the tumor environment.

### Construction of the predictive nomogram

Using the “rms” R package, a nomogram was established to predict the 1–5 year survival rate of HNSCC patients. The calibration curve is used to evaluate the prediction ability of nomogram. The closer to the 45° line, the better the prediction ability. Then, the independent prognostic factors were compared by decision curve analysis (DCA) to explore the difference of the combined nomogram in clinical benefit.

### Genomic variance analysis

In order to identify the genomic variation, the distribution of all somatic mutant genes was compared. Somatic mutation data were downloaded from the Genomic data commons (GDC) database (https://portal.gdc.cancer.gov/). A comparison of tumor mutation burden (TMB) was conducted between the high-risk group and low-risk group.

### Statistical analysis

All the statistical analyses of this study were performed using R software. The Wilcoxon test was used to selected DEGs of the two groups. Kaplan–Meier survival analysis and the Cox regression model were used for univariate survival analysis and multivariate survival analysis, respectively. Wilcoxon test was used to determine whether there was a difference in the proportion of somatic mutations. Bilateral P < 0.05 was considered significant.

## Results

### Construction the prognostic signature of cuproptosis and copper metabolism–related genes and validating its predictive performance

**Figure 1:**
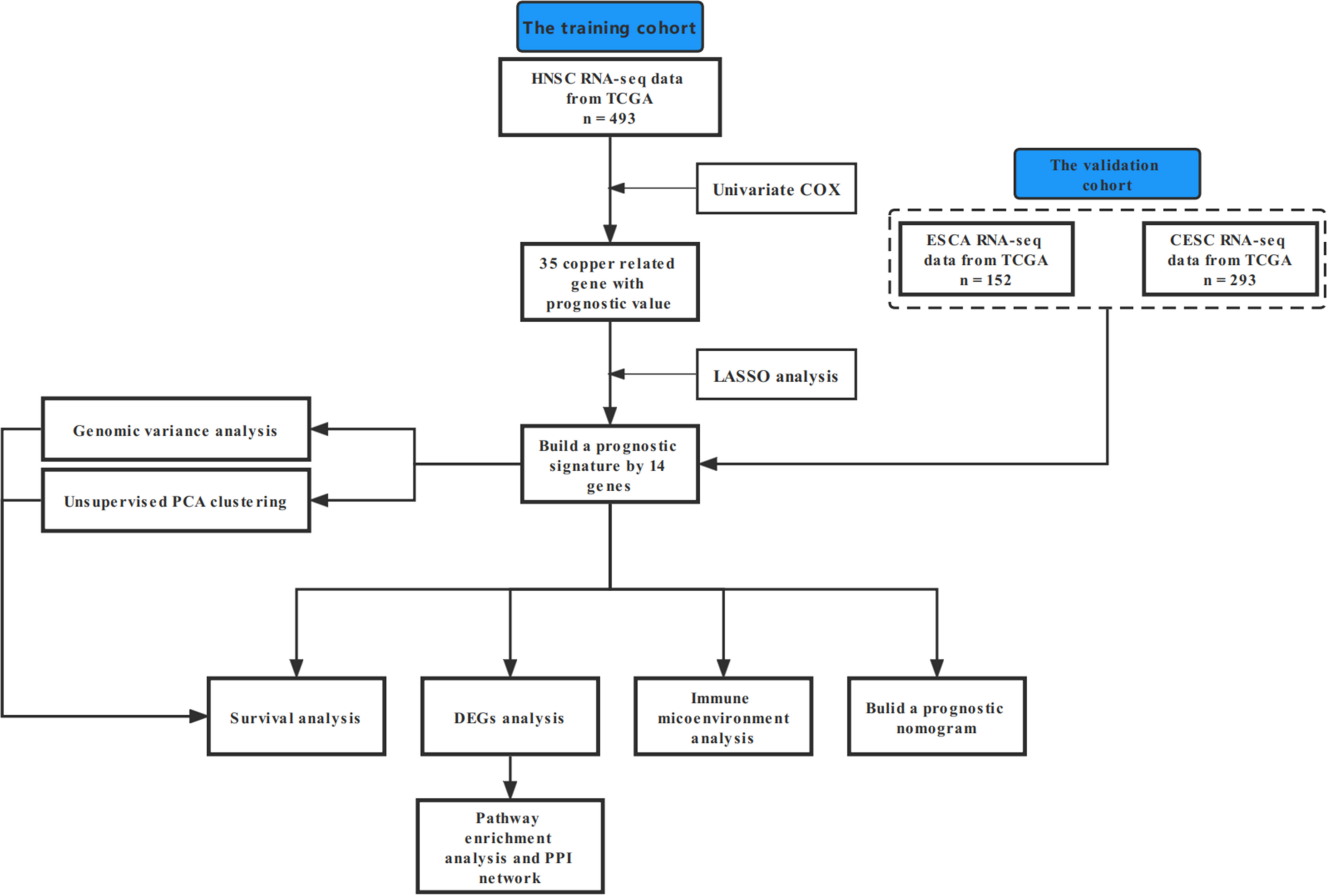
A flowchart outlining the schematic design of a study.

**Figure 2:**
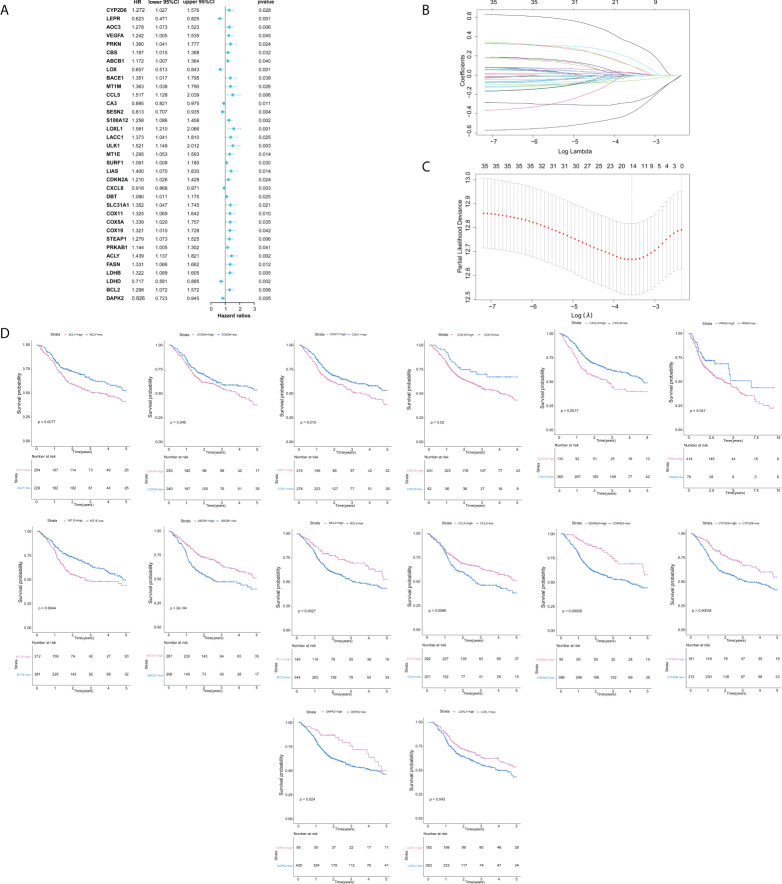
Construction of cuproptosis and copper metabolism–related gene prognostic signature. **(A)** Cuproptosis and copper metabolism–related genes of Cox analysis. **(B, C)** Least absolute shrinkage and selection operator analysis was performed to select the optimum genes to construct a 14-gene signature. **(D)** K-M curve of 14 genes.

In this study, our bioinformatics analysis is based on the workflow shown in **Figure 1**. A total of 546 samples from the TCGA database were used; 44 normal samples, incomplete prognostic information, and OS less than 30 days were excluded; and 493 cancer samples were eventually included in the study. To establish a novel prognostic model, 35 genes related to prognosis were selected from 139 genes by univariate Cox analysis, and then, the appropriate genes related to cuproptosis and copper metabolism were selected by LASSO regression analysis (**Figure 2A** and **Additional Figure 1**). Finally, based on the optimal value of λ, a 14-gene signature was identified (**Figures 2B, C**). **Figure 2D** shows the K-M curve of each gene.

**Figure 3:**
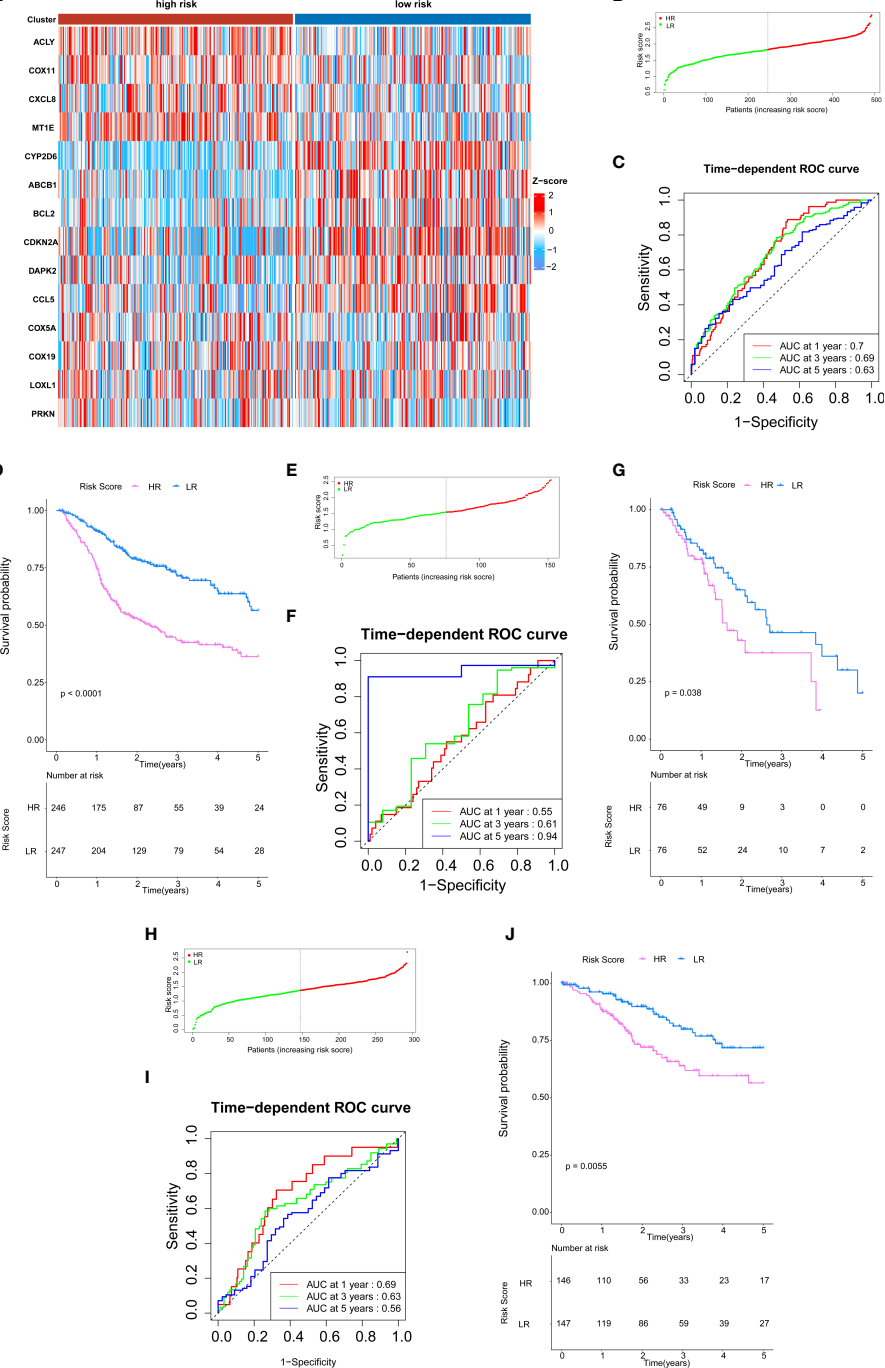
Validation of cuproptosis and copper metabolism–related gene prognostic signature. **(A)**Heat map representing the expression level of the prognostic genes based on the high-risk group and the low-risk group of head and neck squamous cell carcinoma (HNSCC) patients. **(B, E, H)** Association between the risk score and the distribution of high- (red) and low- (green) risk groups of HNSCC patients **(B)**, esophageal cancer (ESCA) patients **(E)**, and CESC patients **(H)**. **(C, F, I)** The ROC curves for evaluating the prognostic value by the 14-gene signature in the TCGA cohort of HNSCC patients **(C)**, ESCA patients **(F)**, and CESC patients **(I)**. **(D, G, J)** The overall survival (OS) rates of different groups of HNSCC patients **(D)**, ESCA patients **(G)**, and CESC patients **(J)**.

As mentioned above, 14 gene-based prognostic signatures were used to compute the risk score for each sample (**Figures 3A, B**). The formula is as follows: risk score = (-0.3847 × expression of *CYP2D6*) + (0.1679 × expression of *PRKN*) + (-0.2781 × expression of *ABCB1*) + (-0.0177 × expression of *CCL5*) + (-0.0066 × expression of *LOX*L1) + (0.0252 × expression of *MT1E*) + (-0.0554 × expression of *CDKN2A*) + (0.1500 × expression of *CXCL8*) + (0.3308 × expression of *COX11*) + (0.0942 × expression of *COX5A*) + (0.0945 × expression of *COX1*9) + (0.0222 × expression of *ACLY*) + (-0.0005 × expression of *BCL2*) + (-0.0047 × expression of *DAPK2*). All patients were grouped into a high-risk group (n=246) or a low-risk group (n=247) according to the median risk score. Kaplan–Meier survival analysis indicated that the OS of high-risk groups was markedly lower than that of low-risk groups (Kaplan–Meier survival analysis P < 0.0001, **Figure 3D**). The ROC curve showed that the risk score development in our present study had a good predictive value of 0.70, 0.69, and 0.63 for 1-year AUC, 3-year AUC, and 5-year AUC, respectively (**Figure 3C**). Therefore, according to the survival curve, we finally determined that 14 genes related to cuproptosis and copper metabolism were significantly correlated with prognosis: *ACLY*, *COX5A*, *COX11*, *COX1*9, *CXCL8*, *PRKN*, and *MTIE* were negatively correlated with OS, while *ABCB1*, *BCL2*, *CCL5*, *CDKN2A*, *CYP2D6*, *DAPK2*, and *LOXL1* were positively correlated with OS. Similarly, PCA confirmed that these 14 genes have implications for OS prediction (**Additional Figure 1**). In conclusion, the above results suggest that the model composed of cuproptosis and copper metabolism–related genes may be a credible and valuable indicator for predicting the prognosis of HNSCC.

We intended to select the available HNSCC sample cohort to verify the above results. However, the model genes were incomplete in any other HNSCC cohort of Gene expression omnibus (GEO) databases. Therefore, we selected other cohorts for validation. All three malignancies contain an epithelial origin (1, 20, 21). On one hand, ESCA and HNSCC have high homology; they have a similar metastatic behavior and have common internal resistance to conventional systemic therapy (22). On the other hand, esophagus is a high-risk site for HNSCC patients to develop secondary primary malignant tumors (23, 24). Based on these correlations, we selected the available cohorts of ESCA samples from the TCGA database for verification, including 152 patients with ESCA (**Figures 3E–G**). For HNSCC and CESC, studies have shown that their immune activation transcriptome has a certain correlation (25). Therefore, we also validated our model in the CESC cohort (**Figures 3H–J**). The results of this validation queue were consistent with the results we obtained before, which showed that it had excellent reliability and repeatability.

### Differentially expressed gene identification and bioinformatics analysis

**Figure 4:**
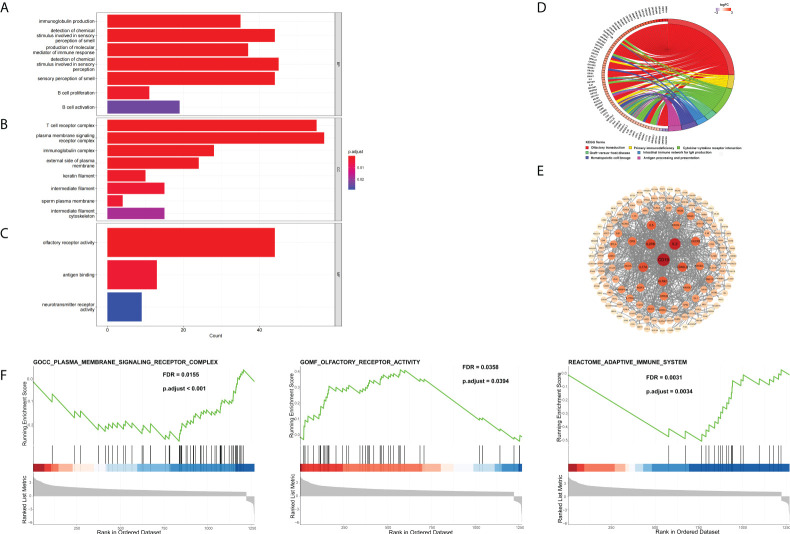
DEG Identification and bioinformatics analysis. **(A–D)** The enriched GO **(A)** BP, **(B)** CC, and **(C)** MF terms as well as **(D)** KEGG pathways. GO, gene ontology; KEGG, Kyoto Encyclopedia of Genes and Genomes; DEG, differentially expressed gene; BP, biological process; CC, cellular component; MF, molecular function. **(E)** The PPI network downloaded from the STRING database indicated the interactions among the candidate genes. **(F)** Gene set enrichment analysis.

To further investigate the possible biological functions of 14 genes related to cuproptosis and copper metabolism, we used the “clusterProfiler” package for GO annotations and KEGG pathway enrichment analysis. **Figure 2** lists the enriched GO terms and KEGG pathways. DEGs were significantly enriched in immunoglobulin production, the detection of chemicals, stimulus involved in sensory, olfactory pathways, T-cell receptor complexes, and plasma membrane signals (**Figures 4A, B**). The top three significantly enriched molecular function terms include olfactory receptor activity, antigen binding, and neurotransmitter receptor activity (**Figure 4C**). In addition, KEGG analysis revealed that the significant enrichment pathway of these DEGs was the olfactory transduction pathway (**Figure 4D**). The PPI network of DEGs was acquired through the application of the online STRING tool. The results showed that the CD19 molecule (CD19) was the most important gene (**Figure 4E**). The gene coding protein is a member of immunoglobulin gene superfamily. Its coding protein is cell surface protein, and its expression is limited to B-cell lymphocytes. Moreover, we used GSEA to explore the potential biological functions of 14 genes involved in cuproptosis and copper metabolism. The results of GSEA confirm these findings (**Figure 4F**).

### Analysis of tumor immune microenvironment

**Figure 5:**
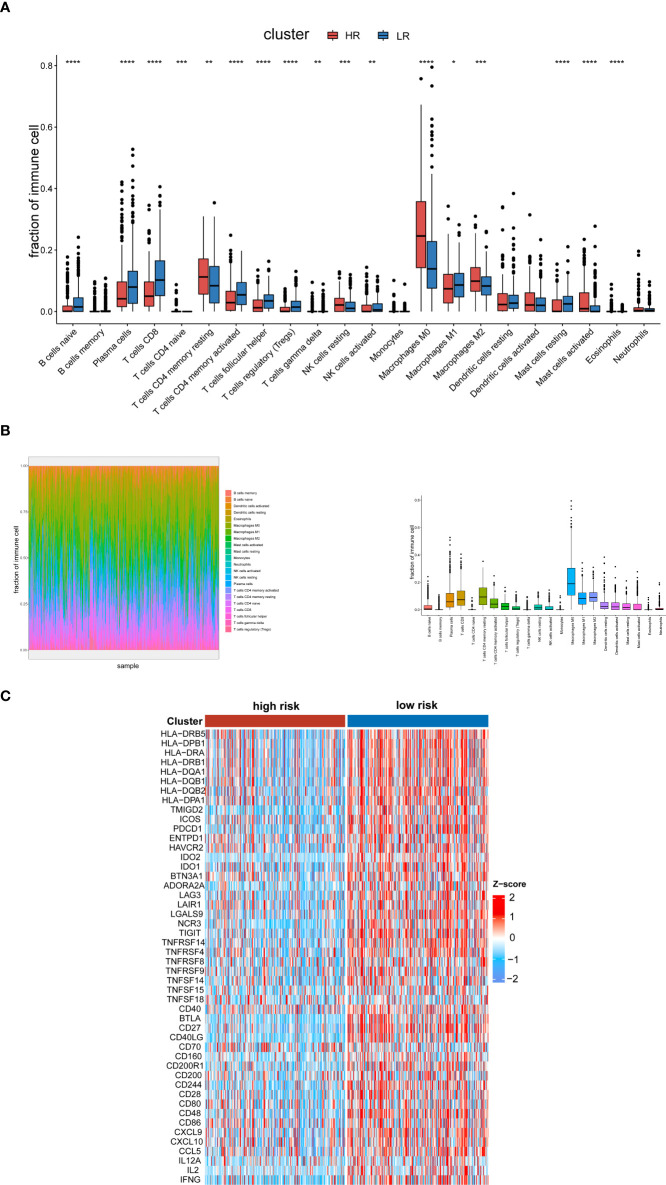
Analysis of the tumor immune microenvironment. **(A)** Analysis of the abundance of immune cells *via* the CIBERSORTx algorithm based on the TCGA cohort. **(B)** The characteristics related to the immune landscape of two groups *via* the CIBERSORTx algorithm. **(C)** Heat map of human leukocyte antigen, immune checkpoint, and inflammation-related promotion in the low- and high-risk groups in the TCGA databases. The p-values were shown as: *p < 0.05; **p < 0.01; ***p < 0.001. ****p < 0.0001.

Through DEG analysis, we found that these genes play a role in immune-related pathways. As a result, we explored the connection between the cuproptosis and copper metabolism–related signature and the tumor immune microenvironment. We used the CIBERSORTx algorithm and xCell to compare the distribution of immune cells in different groups. It was observed that M2 macrophages, naïve CD4 T cells, resting memory CD4 T cells, resting NK cells, activated mast cells, eosinophils, M0 macrophages, cancer cells, and lymph vessels were more abundant in the high-risk group, while Th17 cells, DC, naïve B cells, plasma cells, CD8 T cells, follicular helper T cells, activated NK cells, activated memory CD4 T cells, M1 macrophages, and resting mast cells were more abounding in the low-risk group (**Figure 5A**, **Additional Figure 2**). We also displayed the characteristics related to the immune landscape of the two groups (**Figure 5B**, **Additional Figure 2**). Immune-related functions such as human leukocyte antigen, immune checkpoint, and inflammation-related promotion between the two groups were usually more significant in low-risk populations (**Figure 5C**).

### Establishment and evaluation of the predictive nomogram

**Figure 6:**
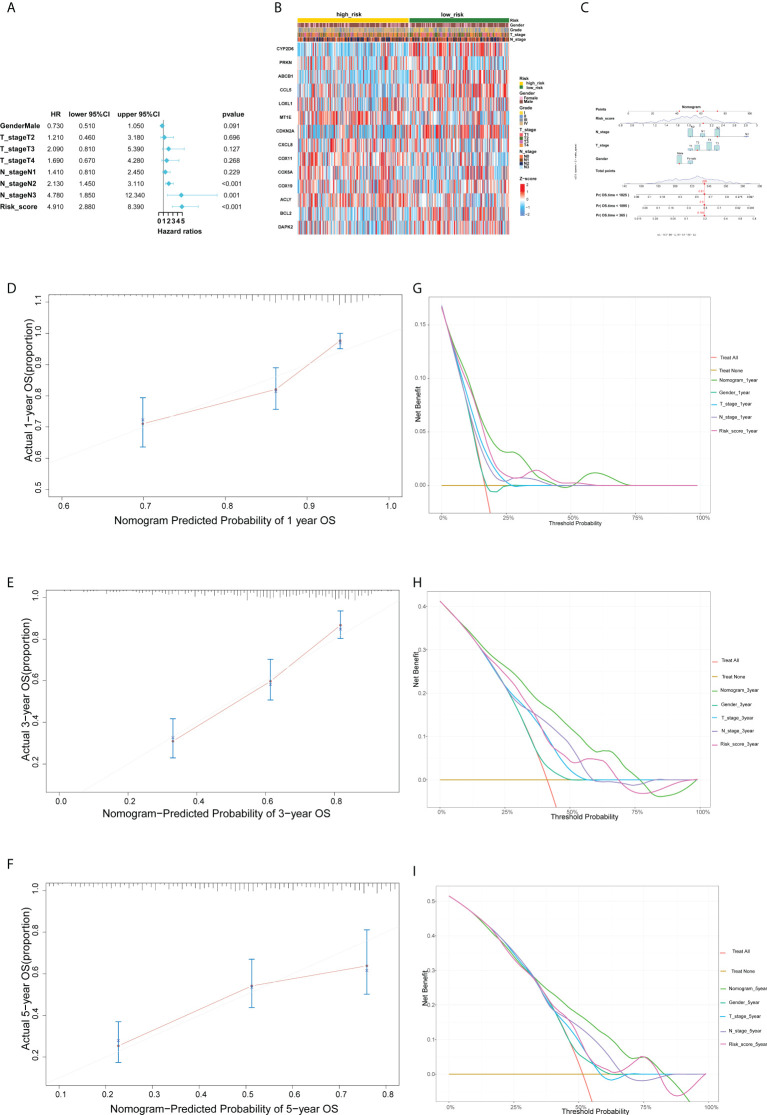
Establishment and evaluation of the predictive nomogram.**(A)**Multivariate Cox regression analysis of the clinicopathological features in the TCGA cohort. **(B)** Heat map for cuproptosis and copper metabolism–related gene prognostic signature and clinicopathological manifestations. **(C)** A nomogram for both clinic-pathological factors and prognostic cuproptosis and copper metabolism–related genes. **(D–F)** Plots depict the calibration of nomograms based on the risk score in terms of the agreement between predicted and observed 1-year **(D)**, 3-year **(E)**, and 5-year **(F)** outcomes in the TCGA cohort. **(G, H, I)** Decision curve analyses of the nomograms based on OS in two cohorts for 1 year **(G)**, 3 years **(H)**, and 5 years **(I)**.

Multivariate Cox regression was used for determining whether the risk characteristics were independent prognostic factors (**Figure 6A**). We analyzed the heat map of the association between the prognostic characteristics of cuproptosis and copper metabolism related genes and clinicopathological manifestations, which can be used for the clinical management of HNSCC patients (**Figure 6B**). To further enhance the predictive ability, a predictive nomogram ground on the integration of the risk score and pathological stage was constructed in the TCGA cohort (**Figure 6C**). The calibration curve of the nomogram showed that the standard curves for predicting and observing 1-year, 3-year, and 5-year results in the cohort were fairly consistent (**Figures 6D–F**). In addition, we used DCA analysis to evaluate the predicted values of nomogram for the TCGA cohort 1, 3, and 5 years in clinical decision-making (**Figures 6G–I**).

### Genomic variance analysis in the TCGA cohort

**Figure 7:**
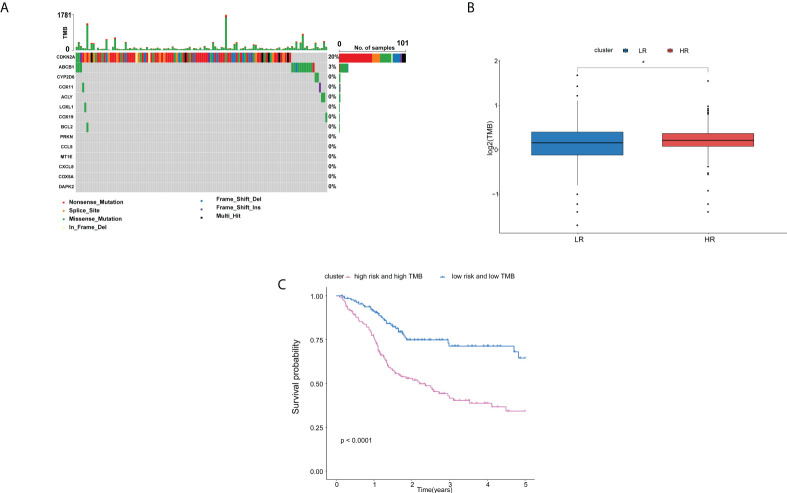
Analysis of the differences of genomics between high- and low-risk groups in the TCGA cohort.**(A)** Waterfall chart depicting the differential signature genes in somatic mutations of the HNSCC. **(B)** Boxplots displaying the TMB of the high-risk patients compared to low-risk patients. **(C)** OS rates in low-risk patients with low TMB were higher than those in high-risk patients with high TMB. Adjusted P-values were shown as: ns, not significant; *P < 0.05.

In order to explore whether the difference of the copper metabolism gene expression between the two groups is related to gene variation, we conducted genomic variance analysis. We found that *CDKN2A* and *ABCB1* had a high mutation probability in HNSCC (**Figure 7A**). The results showed that TMB in high-risk patients was higher than that in low-risk groups (P < 0.05, **Figure 7B**). The OS of patients with low risk and low TMB is higher than that of patients with high risk and high TMB (P < 0.0001, **Figure 6C**).

## Discussion

In this study, we divided HNSCC into two risk subgroups with different TME characteristics based on 14 genes related to cuproptosis and copper metabolism. The characteristics of subgroup survival analysis were also well displayed according to clinical characteristics such as the tumor grade and stage. These results show the cuproptosis and copper metabolism-related genes signature can be considered as a validated dependable model for HNSCC.

All genes are involved in copper homeostasis and transport, oxidative stress, and the TCA cycle (26–34). *COX11* is a copper transfer factor, while *COX1*9 facilitates the transfer of copper (26). It was found that among multiple MT subtypes, *MT1E* was significantly upregulated in transcription after copper treatment, and its expression change can be used as an indicator of intracellular copper ion variation (6). The interaction between copper chelate and *ABCB1* inhibited its mediated transport and downregulated the expression of *ABCB1* (27). The genetic variation of *PRKN* is closely related to the pathogenesis of Parkinson’s disease, mainly manifested in mitochondrial dysfunction (35–37). *CYP2D6*, *DAPK2*, *BCL2*, *RANTES*, and *IL-8* are all related to the redox characteristics of copper (29, 34, 38). *LOX* inactivation due to copper metabolism disorders or genetic mutations leads to the dysfunction of connective tissue (39). The enzymatic activity of *ACLY* has important functions in the TCA cycle (40). In addition, *CDKN2A* is a key gene regulating cuproptosis and has a high mutation probability in HNSCC (41).

The enrichment analysis of the two groups displayed that the 14 genes related to cuproptosis and copper metabolism were significantly enriched in immunocytes, tumorigenesis, such as immune response, intermediate filament, B-cell proliferation, and so on (42–46) The correlation analysis of the tumor immune microenvironment revealed that the activation of cells in the high-risk group had the characteristics of “cold tumor”, such as naïve CD4 T cells, resting NK cells, and M0 macrophages. On the contrary, in the low-risk group, Th17 cells, DC, B cells, plasma cells, CD8 T cells, activated memory CD4 T cells, follicular helper T cells, activated NK cells, and M1 macrophage cells were more abundant and had a longer survival time. In addition, we investigated and studied the relevance between cuproptosis and copper metabolism–related genes, immune activity, and immune checkpoints. Our results show that there is a close interaction between copper metabolism and immunity. Immunity may play a crucial part in the occurrence of cuproptosis. Cuproptosis may affect the immune state of HNSCC, thus affecting the occurrence and development of tumor. Previous studies have shown that the imbalance of copper metabolism and the changes of copper protein levels are closely related to cancer (47). The dysfunction of copper metabolic proteins may be the initiation of processes such as the generation of pretransfer niche, immune defense escape, and angiogenesis. However, the potential mechanism of inducing the cuproptosis behavior of tumor cells and enhancing antitumor immune response needs to be further clarified, and our research will help to provide new ideas into the exploration of potential tumor-targeted therapies.

Interestingly, using GO, KEGG, and GSEA enrichment analyses, we found that 14 genes associated with cuproptosis and copper metabolism were significantly enriched in the olfactory transduction pathway. In mammals, copper is highly concentrated in brain regions, including the major organs of olfactory receptors (48). The destruction of copper homeostasis is the basis of neurodegenerative diseases, which can be manifested as olfactory dysfunction (49). Patients with Alzheimer’s disease show serious olfactory defects in the early stage, and olfactory dysfunction may occur in patients with Wilson’s disease with neurological symptoms (48, 50). Recent studies have shown that olfactory receptor–related genes not only act on proprioceptive nerves but also play a crucial role in promoting tissue inflammation and cancer metastasis (51). At the same time, combined with the correlation between copper metabolism and tumor in this study, we speculate that there may be a potential relationship between copper metabolism–related genes and olfactory receptors on tumors. This discovery may offer new ideas for the diagnosis and treatment of HNSCC.

In recent years, the establishment of prognostic models for HNSCC has developed rapidly. Some researchers have developed a prognostic signature of regulatory cell death–related genes in HNSCC (52–54). Compared with ferroptosis, pyroptosis, and apoptosis, cuproptosis is a new concept. In this paper, we preliminarily discussed the role of cuproptosis in HNSCC and constructed a robust correlation model to predict the prognosis of HNSCC patients. Some researchers have paid attention to the TMB, which is related to the immunotherapeutic response of HNSCC (55). Therefore, the prognostic value of TMB in HNSCC researchers has been studied (55, 56). Interestingly, we found that the difference of copper metabolism gene expression in HNSCC patients was related to TMB, and the internal relationship between them remains to be explored. As an immunogenic tumor, the establishment of immune checkpoint–related gene signature is helpful to determine which HNSCC patients can benefit from immunotherapy (57, 58). In this study, we explored the possible role of cuproptosis in the progression of HNSCC from the perspective of immunity. We found that cuproptosis may be connected with the immune status of HNSCC and may thus play a role in the occurrence and development of tumors. The in-depth study of their internal mechanism may help to provide new ideas for tumor immunotherapy.

As with all studies, our study also had limitations. Our bioinformatics analysis identified potential cancer diagnostic genes associated with cuproptosis and copper metabolism, but its accuracy remains to be verified for each HNSCC subtype. In addition, our current research results are obtained from databases, and a series of biological experiments should be conducted to further examine the molecular mechanisms involved in cuproptosis and copper metabolism–related genes that affect the prognosis of HNSCC patients.

## Data availability statement

Publicly available datasets were analyzed in this study. This data can be found here: HNSCC, CESC, ESCA cohort from TCGA database, https://portal.gdc.cancer.gov/.

## Author contributions

SZ and JH conceived and designed the study. HL, SZ, and XL performed the data analysis. SZ, LZ, HL, and YY analyzed and interpreted the results. LZ wrote the original manuscript. SZ and JH reviewed and revised the manuscript. All authors contributed to the article and approved the submitted version.

## Funding

This research was supported by the National Natural Science Foundation of China (grant numbers 81874128 and 82072994); Sun Yat-Sen University Clinical Research 5010 Program (grant numbers 2015018).

## Conflict of interest

The authors declare that the research was conducted in the absence of any commercial or financial relationships that could be construed as a potential conflict of interest.

## Publisher’s note

All claims expressed in this article are solely those of the authors and do not necessarily represent those of their affiliated organizations, or those of the publisher, the editors and the reviewers. Any product that may be evaluated in this article, or claim that may be made by its manufacturer, is not guaranteed or endorsed by the publisher.
