# Supplementary material for: A cuproptosis and copper metabolism–related gene prognostic index for head and neck squamous cell carcinoma
Source: Front Oncol. 2022 Aug 22;12:955336. doi: 10.3389/fonc.2022.955336 (PMC9441563; doi:10.3389/fonc.2022.955336)

Supplementary Material

## Supplementary Figures

**
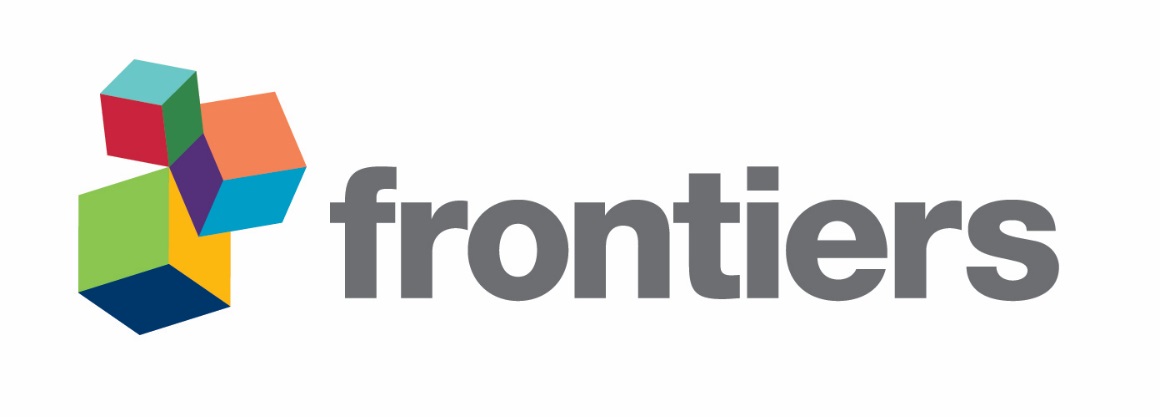
**

**Supplementary Figure 1.** (A) Heatmap of sample clustering at consensus k=2. (B) plot chart Clusters Patients into 2 Clusters. (C) The overall survival rates of different groups of HNSCC patients. (D) Heat map represents the different groups of HNSCC patients.


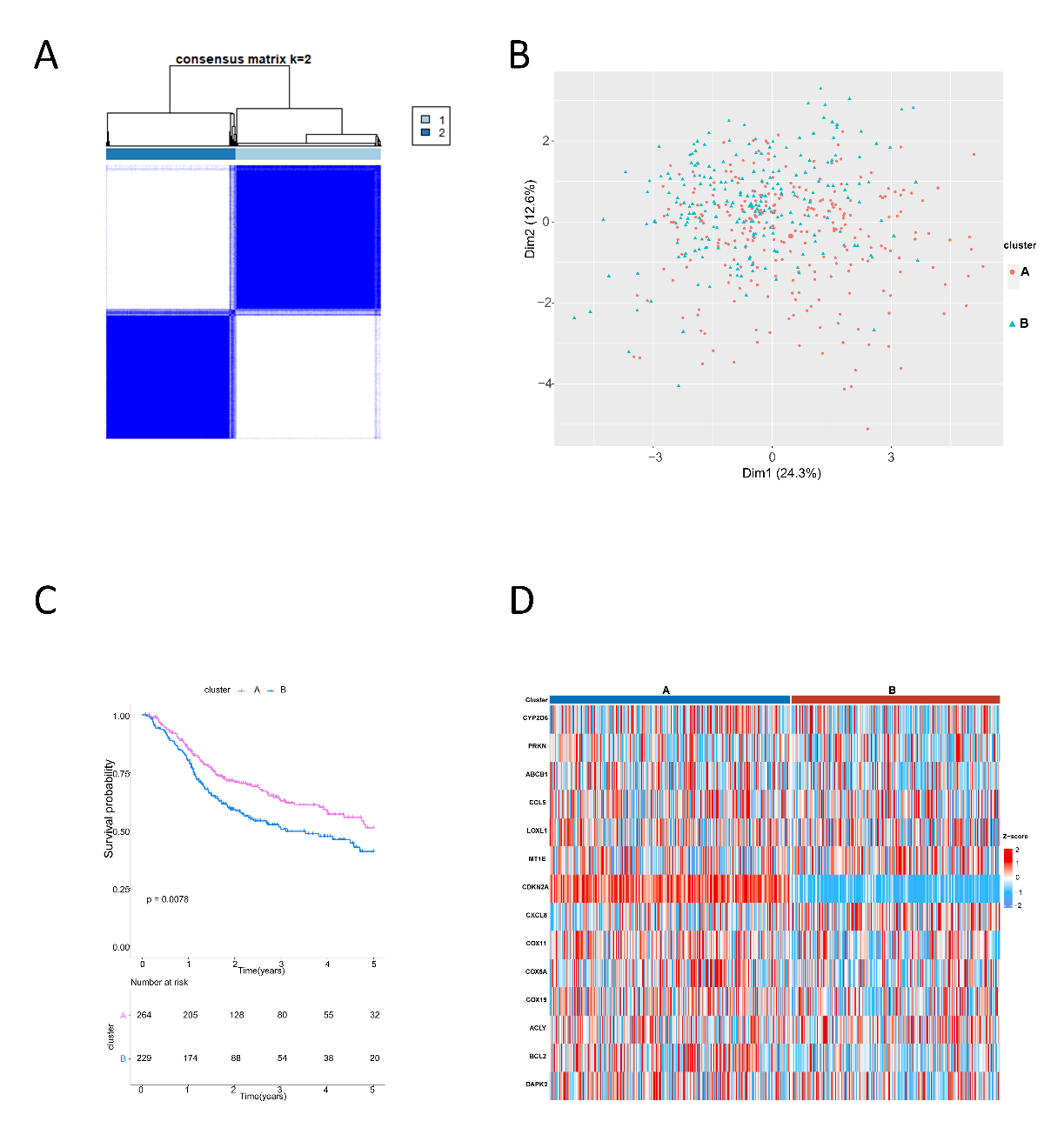


**Supplementary Figure 2.** (A) Analysis of the abundance of immune cells via xCell(B) based on TCGA cohort. (B) The characteristics related to the immune landscape of two groups via xCell. The p values were showed as: *p < 0.05; **p < 0.01; ***p < 0.001.


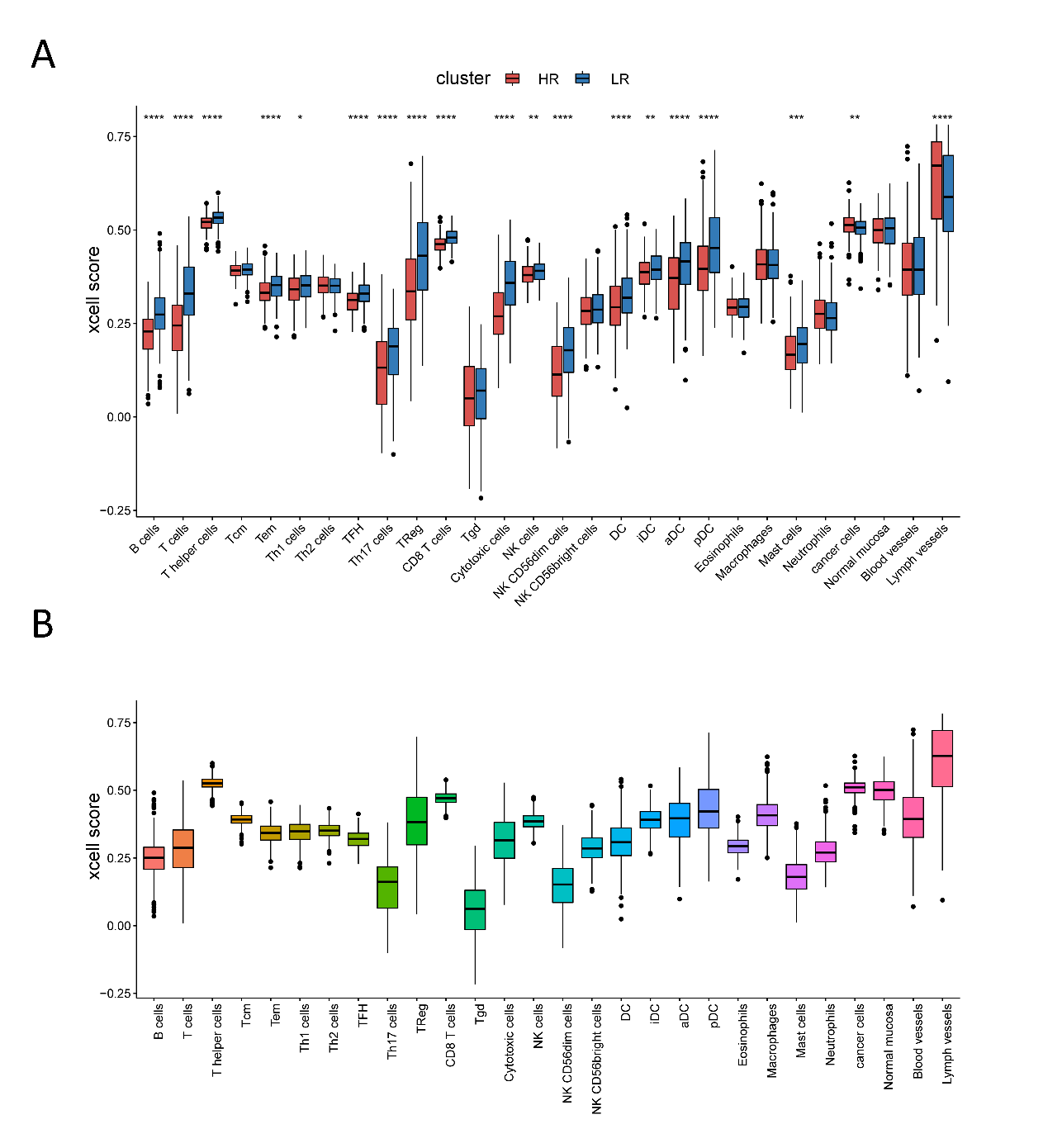

Supplement: Supplementary file 1 [file DataSheet_1.docx]
